# Effect of timing of hip extension assistance during loaded walking with a soft exosuit

**DOI:** 10.1186/s12984-016-0196-8

**Published:** 2016-10-03

**Authors:** Ye Ding, Fausto A. Panizzolo, Christopher Siviy, Philippe Malcolm, Ignacio Galiana, Kenneth G. Holt, Conor J. Walsh

**Affiliations:** 1 John A. Paulson School of Engineering and Applied Sciences, Harvard University, 29 Oxford Street, Cambridge, MA 02138 USA; 2 Wyss Institute for Biologically Inspired Engineering at Harvard, 3 Blackfan Circle, Boston, MA 02115 USA; 3 Sargent College of Health and Rehabilitation Science, Boston University, 635 Commonwealth Ave, Boston, MA 02215 USA

## Abstract

**Background:**

Recent advances in wearable robotic devices have demonstrated the ability to reduce the metabolic cost of walking by assisting the ankle joint. To achieve greater gains in the future it will be important to determine optimal actuation parameters and explore the effect of assisting other joints. The aim of the present work is to investigate how the timing of hip extension assistance affects the positive mechanical power delivered by an exosuit and its effect on biological joint power and metabolic cost during loaded walking. In this study, we evaluated 4 different hip assistive profiles with different actuation timings: early-start-early-peak (ESEP), early-start-late-peak (ESLP), late-start-early-peak (LSEP), late-start-late-peak (LSLP).

**Methods:**

Eight healthy participants walked on a treadmill at a constant speed of 1.5 m · s^-1^ while carrying a 23 kg backpack load. We tested five different conditions: four with the assistive profiles described above and one unpowered condition where no assistance was provided. We evaluated participants’ lower limb kinetics, kinematics, metabolic cost and muscle activation.

**Results:**

The variation of timing in the hip extension assistance resulted in a different amount of mechanical power delivered to the wearer across conditions; with the ESLP condition providing a significantly higher amount of positive mechanical power (0.219 ± 0.006 W · kg^-1^) with respect to the other powered conditions. Biological joint power was significantly reduced at the hip (ESEP and ESLP) and at the knee (ESEP, ESLP and LSEP) with respect to the unpowered condition. Further, all assistive profiles significantly reduced the metabolic cost of walking compared to the unpowered condition by 5.7 ± 1.5 %, 8.5 ± 0.9 %, 6.3 ± 1.4 % and 7.1 ± 1.9 % (mean ± SE for ESEP, ESLP, LSEP, LSLP, respectively).

**Conclusions:**

The highest positive mechanical power delivered by the soft exosuit was reported in the ESLP condition, which showed also a significant reduction in both biological hip and knee joint power. Further, the ESLP condition had the highest average metabolic reduction among the powered conditions. Future work on autonomous hip exoskeletons may incorporate these considerations when designing effective control strategies.

**Electronic supplementary material:**

The online version of this article (doi:10.1186/s12984-016-0196-8) contains supplementary material, which is available to authorized users.

## Background

Exoskeletons have been designed to augment the performance of human locomotion for able-bodied individuals [1–13] or to assist patients affected by different pathologies in rehabilitation and daily activities [14–25]. Positive accomplishments including reductions in energetic cost of walking, have been achieved in the past 2 years by means of autonomous or tethered systems [5–9, 26–30].

Among these, the majority of the assistive devices that achieved a net reduction in metabolic cost during walking (compared to walking without wearing the device) provided assistance only to the ankle joint [6–9]. Only one device provided assistance to both the ankle and hip joints and achieved metabolic reduction (compared to walking with the device unpowered and removing the equivalent weight of the device from the payload) [30]. This might be due to the fact that the ankle contributes significantly to forward propulsion [31] and can be approximated as a single degree of freedom joint. Further, it has been hypothesized that biological power generation at the hip is more costly in terms of metabolic rate [32], since it relies more on the contractile muscle fascicle for power production. In contrast, the ankle joint benefits from the passive elastic properties of the Achilles tendon [32, 33]. Consequently, it has been proposed that providing external power to the hip joint via a wearable robot could provide a larger reduction in metabolic cost than providing the same amount of power at the ankle joint [32].

A number of different research groups have investigated the effects of powering the hip joint via simulation [34–36] or experimental studies [4, 5, 21, 24, 37]. Giovacchini et al. [4] developed an autonomous robotic hip exoskeleton that can deliver flexion-extension torques to the wearer but no detailed biomechanical or physiological evaluation has been presented to date. A study by Lenzi et al. [21] showed that actuating a wearer’s hip with a fraction of the nominal torque profile using a modified treadmill-based lower extremity exoskeleton, can reduce both hip flexor and ankle plantar flexor muscle activation. Lewis and Ferris [37] have reported reduced biological joint torques with a pneumatically powered hip exoskeleton, suggesting that humans alter the net muscle moment at the hip when walking with hip assistance so that the net joint moment is the same regardless of the external actuation under same task constraints. The Stride Management Assist system [5] was designed to increase the walk ratio (step length/cadence) when applying a 3 N · m flexion and extension torque at the hip. With this autonomous hip exoskeleton, the authors reported a reduced metabolic cost of ~7 % compared to walking with the exoskeleton unpowered. Ronsse et al. [24] designed a treadmill-mounted hip exoskeleton controlled by means of adaptive oscillators and tested its effect on metabolic cost of walking. They reported a metabolic reduction of ~18 % compared to walking with the exoskeleton unpowered when providing an assistive torque corresponding to 100 % of the average biological torque produced by the hip during walking. Last, previous work from our group [28] compared the effect of hip extension assistance with multi-joint assistance (ankle plantarflexion and hip extension assistance) during loaded walking on metabolic cost with a tethered multi-joint actuation platform. We found an average reduction of 4.6 % compared to walking with the unpowered soft exosuit when delivering an average peak force of 95 N, resulting in an average peak torque of 16 N · m to the hip joint [28]. However, none of the previous research has studied the biomechanical and physiological effects of different assistive profiles at the hip joint.

Understanding the effects of different hip assistive profiles is a fundamental step in the process of designing assistive devices and controllers that can provide better performance in terms of metabolic cost. While there is promising early work in assisting the hip joint, there is limited literature including studies exploring the effects of the timing and magnitude of assistance in a systematic way as it has been done in studies investigating the ankle joint [6, 7, 38, 39].

Therefore, the goal of the present study was to investigate how onset and peak timings of hip extension assistive profiles affected the positive mechanical power delivered by the soft exosuit and its effect on biological joint power and metabolic cost. Previous studies involving ankle exoskeletons [7, 39] showed the importance of actuation timing as it affects the positive mechanical power delivered to the wearer and the metabolic cost of walking. This is also likely to be a key factor for hip exoskeletons as shown by a recent simulation study [40] exploring the optimal hip retraction timing for assisting the hip joint. Thus, we designed four different assistive profiles with the aim of analyzing the effect of two specific features: i) the onset timing and ii) the peak timing during stance. While the timing was varied and the magnitude of peak force was kept constant, the delivered positive mechanical power varied due to the duration of the assistance and the different hip velocities. Assuming no major kinematic changes imposed by our hip extension assistance (as demonstrated by our previous work [28, 30]), a longer period of assistance (early onset timing) and a better synchronization of actuation with a period of high hip joint velocity can deliver more positive mechanical power to the wearer. Thus, we explored different timings to regulate the amount of positive mechanical power delivered during the swing and stance phase, both of which are related to the metabolic cost of walking [41, 42]. We tested the 4 different assistive profiles on eight healthy participants wearing the soft exosuit. Loaded walking was investigated because it is a common task that puts severe mechanical and physiological challenges on human locomotion, thus representing an interesting gait condition to explore the effect of a lower limb exoskeleton on the users. Also, the ability to carry substantial loads is required in many professions that execute highly physically demanding tasks associated with their gait [43].

## Methods

### Soft exosuit and actuation platform

**Fig. 1:**
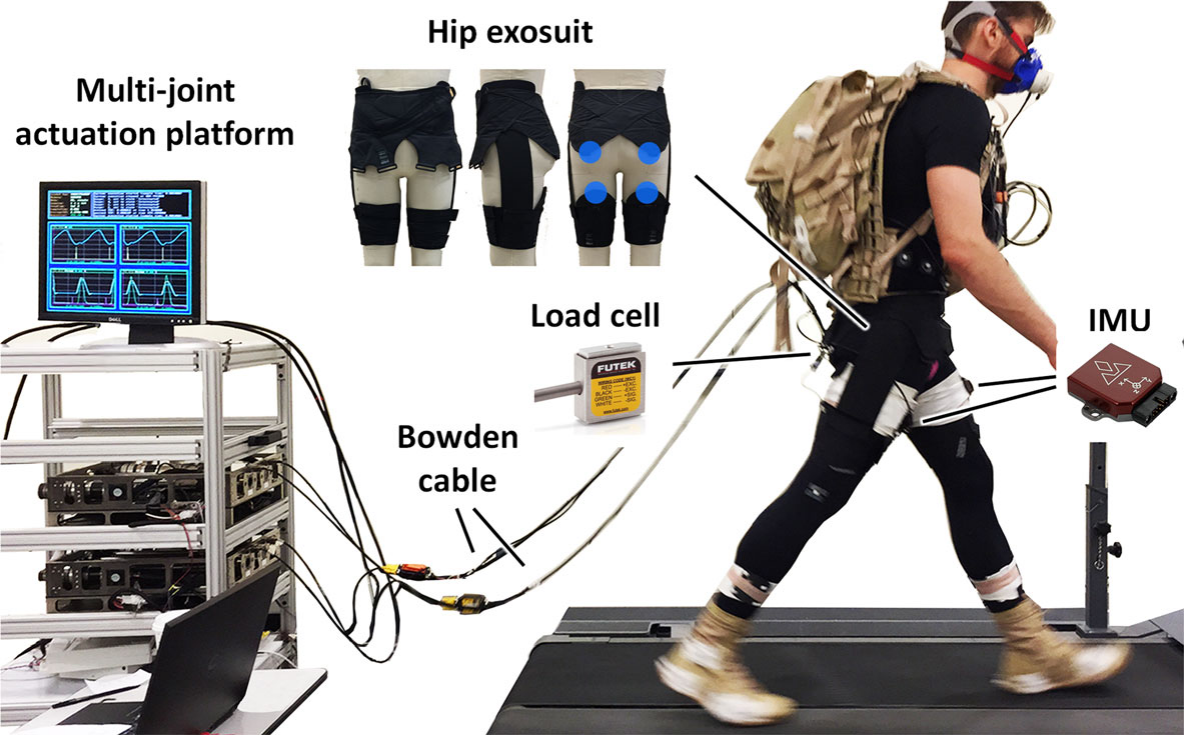
Experimental setup with a participant wearing a soft exosuit that assists hip extension via Bowden cable. The assistive force is transmitted from the multi-joint actuation platform (on the left) to the wearer

For this study, we used a hip soft exosuit and a programmable multi-joint actuation platform that we previously reported in [28, 29, 44]. The textile components of the hip exosuit (Fig. 1) consisted of a spandex base layer (mass: 222 g), a waist belt (mass: 251 g), 2 thigh braces (mass: 2 × 76 g) and 2 inertial measurement unit (IMU) straps (elastic bands that hold IMUs on the anterior part of the thigh; mass: 2 × 35 g). Compared to our previously described hip soft exosuit [28], the components used for this study were constructed with a woven fabric with reinforcement webbing and neoprene was added to the waist belt close to where it interfaced to the iliac crest of the wearer. The multi-joint actuation platform was a tethered system designed to provide biologically inspired torques to multiple joints through Bowden cables either individually or simultaneously [44].

Briefly, in this Bowden cables-based force transmission, the sheath covering the inner cable is fixed to the actuator frame and the inner cable is connected to the actuation carriage on the ball screw. The actuator moves the carriage back and forth to either generate tension forces in the soft exosuit (pulling the cable) or to become fully transparent (feeding out cable so it becomes slack). The other end of the Bowden cable sheath is connected to the attachment point at the bottom of the waist belt and the inner cable is connected to the top center of the thigh brace on the back of the leg (Fig. 1). By pulling the cable, the actuation platform shortens the distance between the 2 attachment points and delivers a controlled force in parallel to the wearer’s hip extension muscle group thus generating an assistive torque around the hip joint.

### Sensing and control

Our system used an IMU-based iterative controller to deliver a consistent force profile in synchrony with the wearer’s individual joint kinematics to accommodate the variability of hip kinetics and kinematics across subjects [29]. One IMU (VectorNav Technologies, Dallas, Texas, USA; mass: 13 g) was attached to the front of each thigh to detect the maximum thigh flexion angle to segment the stride. The algorithm identified the first positive thigh angle peak (corresponding to maximum hip flexion angle) after a negative thigh angle peak (corresponding to maximum hip extension angle) as the maximum hip flexion point. Stride time was measured by the controller as the time between 2 consecutive maximum hip flexion events. Load cells (LSB200, Futek Advanced Sensor, USA; mass: 16 g) were placed in series with the Bowden cables to monitor the delivered force. The simulated trapezoidal position profile of the actuator was calculated based on the desired force, average hip joint kinematics and suit stiffness [29]. This position profile was scaled by the average stride time calculated from the previous 2 steps and commanded to the actuator. The iterative controller then automatically adjusted the offset and magnitude of the position profile based on the measured pretension force and peak force from the previous stride. By continuously correcting the actuator position profile, the desired force profile could be achieved without requiring an accurate initial simulated position profile. This iterative control structure allowed us to robustly control the timing and magnitude of the desired force profiles. Detailed controller performance and evaluation are described in [29].

### Assistive force profiles

**Fig. 2:**
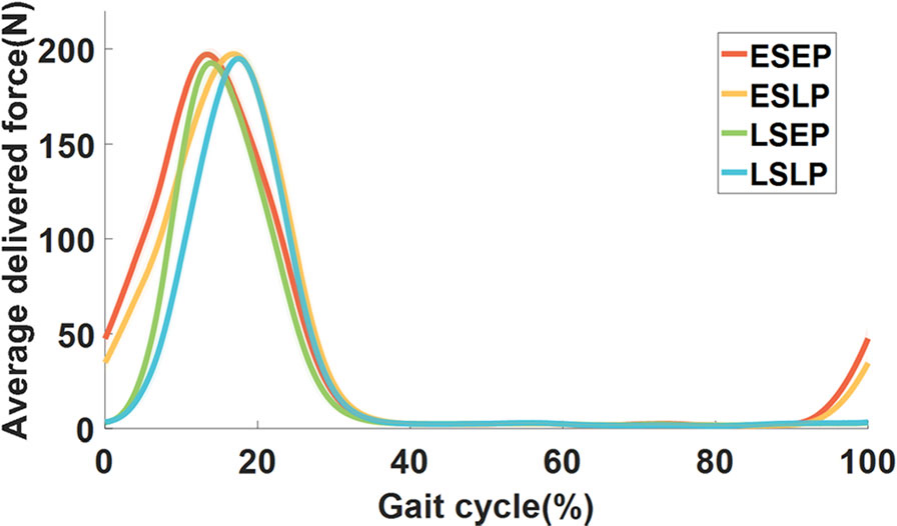
The resulting assistive force profiles with SEM (average from eight subjects) are shown on the right side of the figure. Early-start-early-peak (ESEP), early-start-late-peak (ESLP), late-start-early-peak (LSEP), late-start-late-peak (LSLP) in red, yellow, green, blue respectively

The goal of the present study was to investigate the effect of onset and peak timings for a given level of hip extension assistance with a soft exosuit. In order to compare the onset timing effects, we used 4 different assistive force profiles: 2 profiles with early onset timing to assist hip joint during terminal swing and 2 profiles with late onset timing initiated during early stance (Fig. 2). Early onset timing (around 90 % of the gait cycle) is coincident with the onset of hip extension just prior to heel strike, and the late onset timing (around 0 % of the gait cycle) is coincident with heel strike. Similarly, two of the profiles had early peak timing and the other 2 had late peak timing. Early peak timing (around 13 % of the gait cycle) is approximately coincident with peak hip power [45] and late peak timing (around 17 % of the gait cycle) was chosen as a first exploration to exploit the higher hip velocity present at that point of the gait cycle [45, 46], thus delivering a higher mechanical power while keeping a constant peak force magnitude. Hereinafter, the 4 profiles are referred as early-start-early-peak (ESEP), early-start-late-peak (ESLP), late-start-early-peak (LSEP) and late-start-late-peak (LSLP). The designed and tested onset and peak timing of the profiles are shown in Fig. 2.

### Participants

Eight male healthy participants with no previous experience in walking with the present configuration of the soft exosuit (age 29.8 ± 5.0 year, mass 82.6 ± 5.8 kg, height 1.79 ± 0.05 m, mean ± SD) were recruited for this study. All participants were free from musculoskeletal injuries and other musculoskeletal diseases and provided written informed consent prior to participating in the study. Participants have provided consent for the publication of their images according to the Journal of NeuroEngineering and Rehabilitation policies. The study was approved by the Harvard Medical School Committee on Human Studies.

### Testing protocol

Participants wore the soft exosuit while walking on an instrumented split-belt treadmill (Bertec, Columbus, OH, USA) at a constant speed of 1.5 m · s^-1^ while carrying a 23 kg weighted backpack; these conditions were chosen because representative of a load carrier population such as soldiers and to allow comparisons with previous exoskeletons studies [8].

**Fig. 3:**
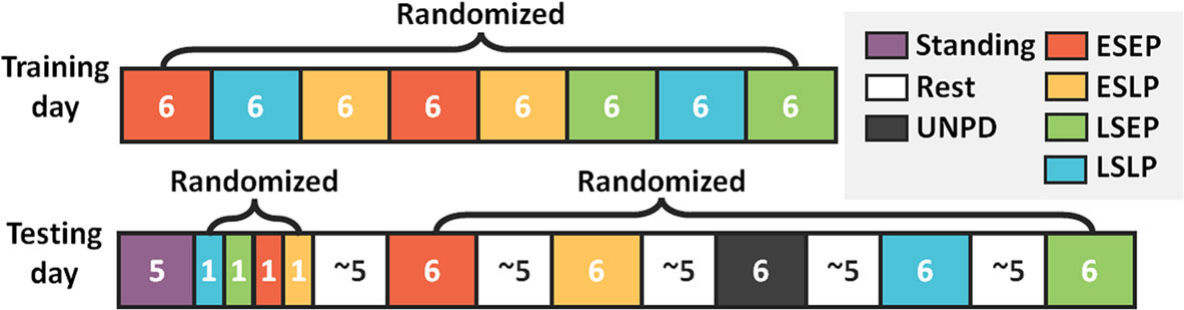
Testing protocol during training and testing session. Numbers in each block represent the duration of each condition: early-start-early-peak (ESEP), early-start-late-peak (ESLP), late-start-early-peak (LSEP), late-start-late-peak (LSLP) and unpowered (UNPD) in red, yellow, green, blue and black respectively

The protocol was split into a training session and a testing session with at least 2 days in between to avoid fatigue effects. During the training session, the participants familiarized themselves with the soft exosuit and the experimental setup. They walked for 8 randomized 6-minute bouts, experiencing each of the 4 different assistive profiles twice. Participants rested between the conditions according to their own requests. At the beginning of the testing session, a 5-minute standing trial was performed to collect steady-state standing metabolic cost. After an initial walking warmup of 4 minutes (1 minute for each assistive profile), the participants took a rest of 5 minutes. Subsequently, they underwent five 6-minute data collection bouts: the 4 assistive conditions and 1 unpowered condition with the device turned off. The 5 walking bouts were randomized to minimize any fatigue, order and learning effects. Adequate rest on an average of 5 minutes was given between walking bouts to allow physical recovery; the training and testing sessions are outlined in Fig. 3. After each condition, participants provided information about their perceptions of assistive conditions on a visual analogue scale (Additional file 1: Table S1).

In the unpowered condition, the participants still wore the soft exosuit as this was connected to the actuation platform. This choice was taken to avoid repositioning the markers used for kinematic analysis and the associated changes in the backpack location which could have led to increased variability. Nevertheless, to assess the difference in metabolic cost between wearing the textile components of the soft exosuit and wearing normal clothing we performed additional testing on 6 participants (age 29.0 ± 4.3 years., mass 75.6 ± 6.4 kg, height 1.79 ± 0.04 m, mean ± SD) on a separate day. After collecting a 4-minute standing trial, participants walked under the same testing conditions (1.5 m · s^-1^ and 23 kg of load) for 2 bouts of 6 minutes wearing the soft exosuit or wearing a pair of pants (mass: 715 g). These 2 conditions were randomized across the participants.

### Data collection and analysis

Kinematic data were collected through 9-camera Vicon optical motion capture system (Oxford Metrics, Oxford, UK; 120 Hz) using 50 markers placed on selected anatomical bony landmarks. Two pairs of additional markers were placed on the right and left proximal and distal attachment points of the Bowden cable. The moment arm of the applied force was calculated continuously across the gait cycle as the normal distance from the line between each pair of markers to the corresponding hip joint center. Ground reaction forces (GRFs) were collected via the instrumented split-belt treadmill. All markers and GRF trajectories were filtered at the same frequency using a zero-lag fourth order low pass Butterworth filter with a 5 –15 Hz optimal cut-off frequency that was selected using a custom residual analysis algorithm which evaluated the difference between the filtered and the unfiltered signals [45] implemented in MATLAB (MATLAB, The MathWorks Inc., USA). Joint angles, joint moments and powers were calculated in the sagittal plane by means of kinematic and inverse dynamics (Visual 3D, C-Motion, Rockville, MD, USA). Joint moments and powers were then normalized by each participant’s body mass. An automatic gait event detection algorithm (Visual 3D, C-Motion, Rockville, MD, USA) was used to determine heel strike in order to define gait cycles. To compute the biological components of net joint moment and power during the powered conditions, the actuation platform was synchronized to the Vicon system using a 5 V signal generated at the beginning of the motion capture data collection. Delivered hip extension moments generated by the soft exosuit during the powered condition were calculated for each participant as the product of the force recorded by the hip load cell and the computed moment arms. Moment arms were defined as the perpendicular distance between the markers on the cable and the respective joint center. The biological joint moments produced during the powered conditions were then calculated by subtracting the moment generated by the soft exosuit at the hip from the net hip joint moment as per [30]. Biological moment was then multiplied by joint velocity to obtain biological power. All data were segmented and normalized to 0 –100 % of the gait cycle. Ten strides per condition collected during the last minute of each condition were used for generating mean kinematic and kinetic data for each individual participant, which were subsequently combined to calculate condition mean data.

Metabolic cost was assessed by indirect calorimetry using a portable gas analysis system (K4b^2^, Cosmed, Roma, Italy), which enabled the measurement of expired gas concentrations and volumes. Carbon dioxide and oxygen rate were averaged across the last 2 minutes (minutes 4 –6) of each walking condition and then used to calculate metabolic rate using the Brockway equation [47]. Net metabolic rate for each condition was obtained by subtracting the standing metabolic power from the walking metabolic power of each condition and then normalizing it by the body mass of each participant. The metabolic reduction was obtained by subtracting the assistive conditions from the unpowered condition. The average metabolic reduction was calculated from the metabolic results of 6 out of 8 subjects. In the 2 subjects not included there were malfunctions in the portable pulmonary gas exchange measurement device during the test which prevented us from using their data.

During the testing session surface electromyographic signals (EMG) from 6 lower limb muscles were measured with a wired system (Delsys, Natick, MA, USA; 2160 Hz). The 6 muscles recorded were: rectus femoris (RF), vastus medialis (VM), gluteus maximum (GM), biceps femoris (BF), soleus (SOL), medial gastrocnemius (MG). Electrodes were placed following guidelines in [48]. EMG signals were band-pass filtered (fourth order Butterworth, cut-off 20 –450 Hz), rectified and low-pass filtered (fourth order Butterworth, cut-off 6 Hz) to obtain an EMG linear envelope. EMG signals were normalized by the average of corresponding EMG peaks recorded during the unpowered condition. Linear envelopes for each muscle group were segmented and normalized to each gait cycle. The same 10 strides per condition used for kinematic and kinetic analysis were used for generating average of muscle activation across each stride, which were subsequently combined to calculate condition mean data.

### Statistical analysis

Repeated measures analysis of variance (ANOVA) was conducted across the 4 powered conditions to assess differences in the positive mechanical power delivered by the soft exosuit. Repeated measures ANOVA including 5 conditions (unpowered, ESEP, ESLP, LSEP, LSLP) were used to verify the effect of assistance on positive biological joint powers, peak flexion and extension joint angles (for hip, knee and ankle), as well as peak and average value of the biological hip extension moment and knee extension moment during the first half of the gait cycle. Additional repeated measures ANOVA were also used to verify the effect of assistance on metabolic cost, spatiotemporal parameters and root mean square (RMS) of muscle activation. If a significant main effect was observed (*p* < 0.050), pairwise comparisons were conducted using Tukey’s honestly significant difference test similar to [49]. Student’s paired *t*-test was performed to assess the differences in net metabolic rate between walking with the textile components of the soft exosuit and walking with standard clothing. Linear regression was used to determine correlations between perceptions scores and metabolic cost. Correlation coefficients (*r*) and significance level (*p* < 0.050), as well as all the statistical analyses were conducted in Matlab (The MathWorks Inc., USA). All the parameters presented in the results section are in the form of mean ± standard error of the mean (SEM).

## Results

Table 1 summarizes the system performance in controlling the onset and peak timings as well as the peak force of the 4 assistive profiles. The average peak of the assistive force was 197.6 ± 0.2 N, which results in an average peak of assistive moment of 30.4 ± 4.7 Nm. The average error between designed and measured onset timing and peak timing were within 1 % of the gait cycle. In all powered conditions, the actuation ended at 35.9 ± 0.6 % of the gait cycle.

**Table 1:** Designed and measured peak force, onset timing and peak timing of the four assistive profiles

	Designed			Measured		
	Peak force (N)	Onset (%)	Peak (%)	Peak force (N)	Onset (%)	Peak (%)
ESEP	200.0	90.0	13.0	198.1 ± 0.1	90.2 ± 0.1	13.3 ± 0.4
ESLP	200.0	90.0	17.0	198.5 ± 0.1	89.5 ± 0.2	17.0 ± 0.4
LSEP	200.0	0.0	13.0	197.7 ± 0.2	0.7 ± 0.1	13.4 ± 0.4
LSLP	200.0	0.0	17.0	196.0 ± 0.2	0.2 ± 0.1	17.9 ± 0.4

Early-start-early-peak (ESEP), early-start-late-peak (ESLP), late-start-early-peak (LSEP), late-start-late-peak (LSLP). Data are mean ± SEM

**Fig. 4:**
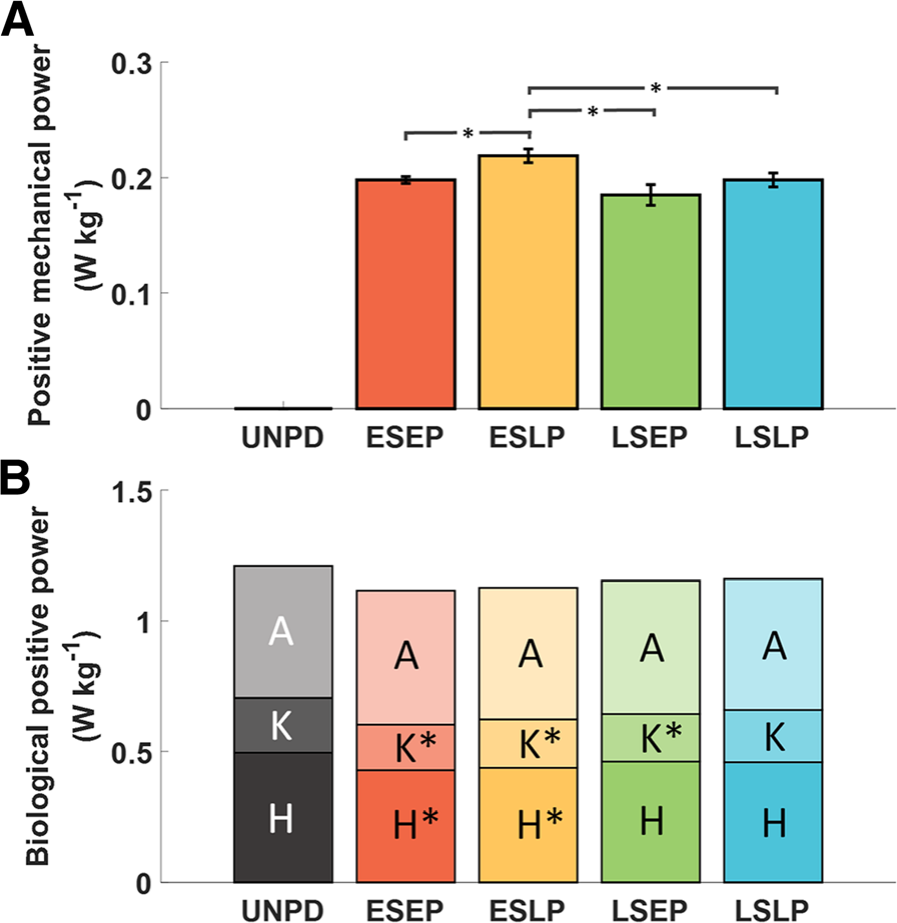
**a** Positive mechanical power delivered by the soft exosuit to both limbs. Data are means ± SEM. The braces with * represent statistically significant differences between two conditions (*p* < 0.050). **b** Average positive biological joint power of both limbs in the unpowered condition (UNPD) and in the powered conditions. The rectangles with letter A, K, H in each bar represent ankle, knee, and hip positive biological joint power. The small panel on the right corner shows the corresponding assistive profile. Early-start-early-peak (ESEP), early-start-late-peak (ESLP), late-start-early-peak (LSEP), late-start-late-peak (LSLP) in red, yellow, green and blue respectively. The * represents statistically significant differences with the unpowered condition (*p* < 0.050)

The positive mechanical power delivered by the soft exosuit to both limbs in the different conditions was 0.198 ± 0.003 W · kg^-1^ (ESEP), 0.219 ± 0.006 W · kg^-1^ (ESLP), 0.185 ± 0.009 W · kg^-1^ (LSEP), 0.198 ± 0.006 W · kg^-1^ (LSLP), as shown in Fig. 4a. ESLP delivered a significantly higher positive mechanical power with respect to the other powered conditions (ESEP; *p* = 0.016, LSLP; *p* = 0.020, and LSEP; *p* < 0.001).

**Fig. 5:**
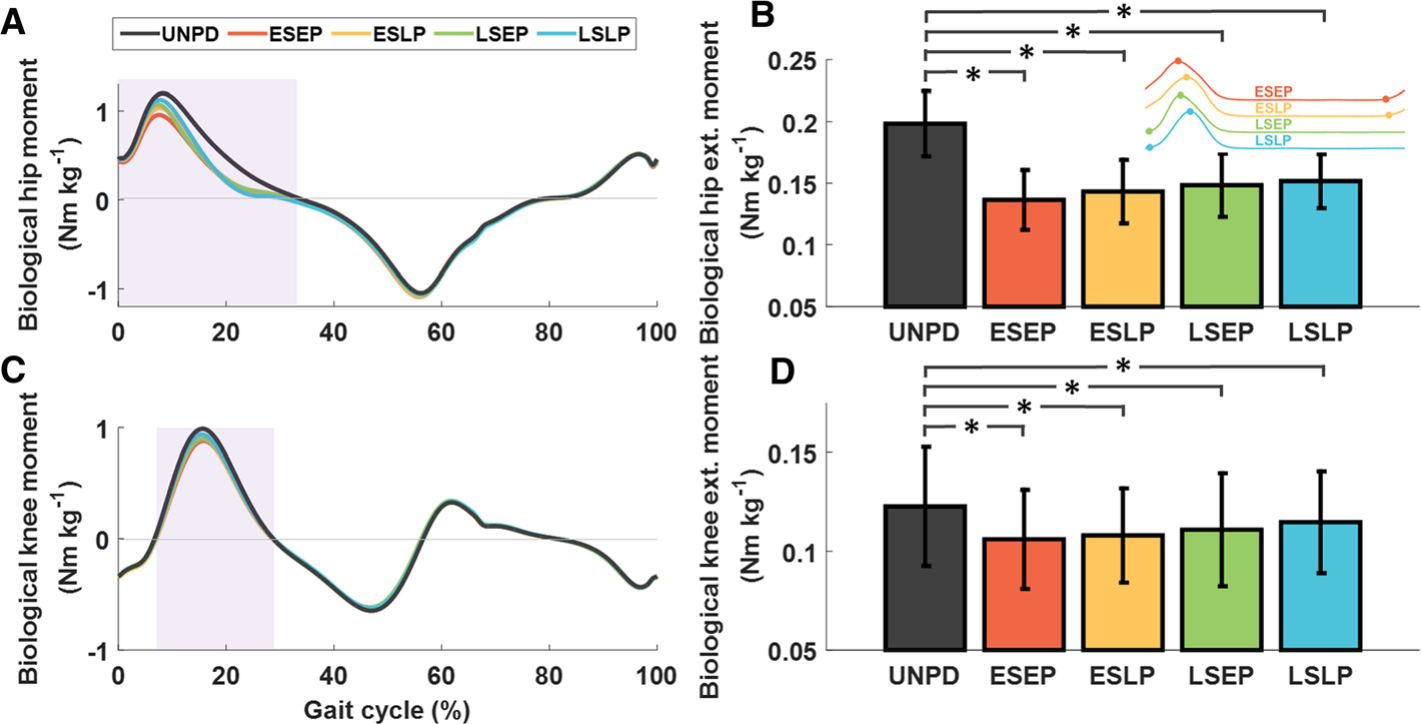
**a**, **c** Average hip and knee biological joint moments of both limbs in the unpowered condition (UNPD) and in the powered conditions plotted versus gait cycle percentage. **b**, **d** Average biological knee and hip extension moment during the first half of the gait cycle (average over the shaded area in **a** and **c**). The small panel on the right corner shows the corresponding assistive profile. Early-start-early-peak (ESEP), early-start-late-peak (ESLP), late-start-early-peak (LSEP), late-start-late-peak (LSLP) in red, yellow, green, blue respectively. The braces with * represent statistically significant differences with the unpowered condition (*p* < 0.050)

In the ESLP condition, the average positive biological knee power was reduced by 0.02 ± 0.01 W · kg^-1^ (*p* = 0.007) and the average positive biological hip power was reduced by 0.06 ± 0.03 W · kg^-1^ (*p* = 0.007) with respect to the unpowered condition. In the ESEP condition, the average positive biological knee power was reduced by 0.04 ± 0.01 W · kg^-1^ (*p* < 0.001) and the average positive biological hip power was reduced by 0.07 ± 0.02 W · kg^-1^ (*p* = 0.002) compared to the unpowered condition. In the LSEP condition, the average positive biological knee power was reduced by 0.03 ± 0.01 W · kg^-1^ (*p* = 0.002). No changes in the average positive biological joint power were observed at the ankle joint (*p* = 0.584). Average positive biological joint power results are presented in Fig. 4b. Average value of the biological hip extension moment was reduced in all the powered conditions (*p* < 0.001) and peak knee extension moment during the first half of the gait cycle was reduced in the ESEP and in the LSEP conditions (*p* = 0.009 and *p* = 0.044, respectively), Fig. 5. No changes in average positive joint moment were observed in the ankle joint (*p* = 0.432).

**Fig. 6:**
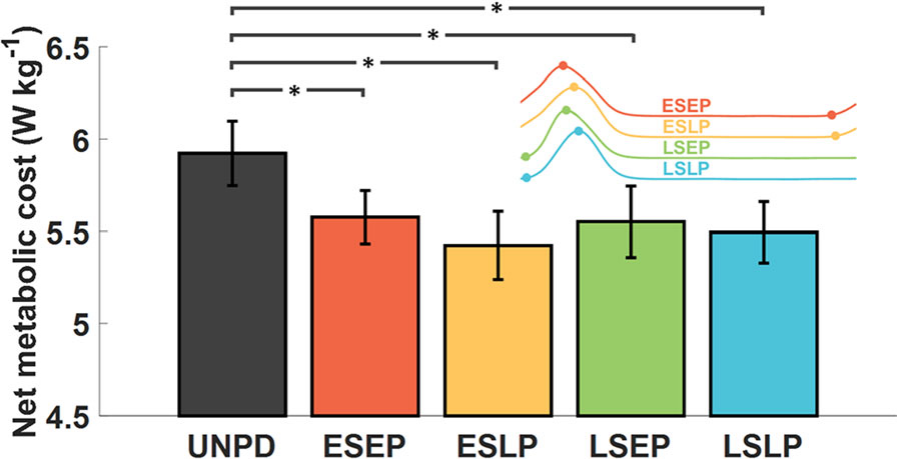
Net metabolic cost reported in the unpowered condition (UNPD) and in the powered conditions. Data are means ± SEM. The small panel on the right corner shows the corresponding assistive profile. Early-start-early-peak (ESEP), early-start-late-peak (ESLP), late-start-early-peak (LSEP), late-start-late-peak (LSLP) in red, yellow, green and blue respectively. The braces with * represent statistically significant differences with the unpowered condition (*p* < 0.050)

Standing with the 23 kg load required 1.52 ± 0.08 W · kg^-1^ and walking in the unpowered condition was 5.92 ± 0.18 W · kg^-1^. All powered conditions significantly reduced metabolic cost compared to the unpowered condition (*p* < 0.015) but no significant differences were found among the powered conditions (Fig. 6). Reductions in metabolic rate in the powered conditions were 0.35 ± 0.10 W · kg^-1^ (ESEP), 0.50 ± 0.05 W · kg^-1^ (ESLP), 0.37 ± 0.08 W · kg^-1^ (LSEP), 0.42 ± 0.11 W · kg^-1^ (LSLP). These values correspond to relative reductions of 5.7 ± 1.5 %, 8.5 ± 0.9 %, 6.3 ± 1.4 % and 7.1 ± 1.9 % respectively. A small, not statistically significant difference (0.10 ± 0.14 W · kg^-1^, *p* = 0.509) in the net metabolic cost was found between walking with the textile components of the soft exosuit and with a pair of regular pants (5.62 ± 0.20 W · kg^-1^ and 5.52 ± 0.13 W · kg^-1^, respectively).

There were no significant differences in spatiotemporal parameters across the conditions (*p* ≥ 0.120, Additional file 1: Table S2). There were no significant changes in peak extension and flexion in all joint angles (*p* ≥ 0.088). Also, no significant changes were found in RMS of muscle activations across the conditions (*p* ≥ 0.097). There was no significant correlation between perception scores and metabolic reduction (*p* ≥ 0.810, *r* ≤ 0.045). Questionnaire results are presented in Additional file 1: Table S1.

## Discussion

The aim of this study was to investigate how the onset and peak timing of hip extension assistance for a given amount of peak force would affect the positive mechanical power delivered by the soft exosuit and its effect on biological joint power and metabolic cost during loaded walking. Results demonstrated that the proposed controller can deliver consistent profiles to the hip joint during loaded walking. The highest amount of positive mechanical power was delivered in the ESLP condition, indicating that a long duration of assistance (early onset timing) together with exploiting higher hip velocity (later peak timing) is a favorable strategy in order to deliver more positive mechanical power to the wearer.

Biological joint power was reduced at the hip (ESEP and ESLP) and at the knee (ESEP, ESLP and LSEP). It is reasonable to hypothesize that these reductions in biological joint power led, at least in part, to the metabolic reduction, similar to the findings of previous work on an ankle-only and on a multi-joint exoskeleton [9, 28, 30]. Further, the ESLP condition, which achieved the highest metabolic reduction, was one condition that reduced biological joint power for both the knee and the hip joints. This result may indicate that an additional metabolic saving is obtained when the external assistance alters the mechanics of non-assisted joints, thus favorably tuning the musculoskeletal system as a whole [6, 9, 28] rather than acting on a specific muscle group. The reduction of the biological joint moment during the first half of the gait cycle in the assistive profiles may also have contributed to the metabolic reduction. This is because the biological joint moment is proportional to the muscle forces, and the cost of muscle force production accounts for ~50 % of the metabolic cost of transport in humans [50]. Recent exoskeleton studies have also proposed a reduction in joint moment as an explanation for the decreased metabolic cost [6, 51].

Metabolic cost was significantly reduced in all the powered conditions compared to the unpowered condition, confirming the efficacy of the hip assistance in reducing the metabolic cost of loaded walking (Fig. 6). Nevertheless, the lack of statistically significant differences between the powered conditions prevents conclusions on the best assistive profile to optimize the metabolic expenditure.

No significant differences were found in muscle activation, similar to our previous work with a hip actuated soft exosuit [28]. Due to the different factors affecting muscle force output [52], the reduction on hip biological joint moment during the first half of the gait cycle does not only depend on muscle activation. It is likely that the lower moment may be explained by a combination of muscle length, velocity and interaction with the tendon [53]. Moreover, all the hip extensors contribute to the joint moment, while only BF was evaluated in this study. Therefore, a reduced muscle activation may not have been observed due to the small magnitude changes of each muscle. Further studies would be required to provide a more mechanistic explanation. For the subjective measurements collected in the questionnaire, we found there were no correlations between perceived assistance scores and metabolic cost. This finding suggested that it is hard to estimate metabolic cost reductions during hip assistance with a simple subjective measurement.

Last, it is worth noting a limitation in our experiment. During the testing protocol we did not include a direct comparison of an exosuit powered and a no exosuit condition, instead we compared the exosuit powered *vs* unpowered separately from exosuit unpowered to normal walking. The reason we compared only powered *vs* unpowered was for the practical considerations outlined in the methods section. Separate testing to evaluate the effect of wearing the suit unpowered was performed later to better understand the metabolic penalty when wearing the suit components compared to walking with a pair of pants. We found a small increase in metabolic rate (0.10 W · kg^-1^) but not statistically significant.

## Conclusions

This study provided insight on how to manipulate the actuation timings to regulate the positive mechanical power delivered by a tethered soft exosuit assisting hip extension. Starting the assistance at terminal swing with a later peak force timing under the same magnitude of peak force allowed the soft exosuit to deliver the highest amount of positive mechanical power. This resulted in reductions in biological hip and knee power, perhaps representing a more beneficial strategy for lowering the metabolic cost. Further, reduced metabolic cost and average of the biological hip extension moment during the first half of the gait cycle were also reported for all the assistive profiles investigated in the present study, although no significant differences were reported between powered conditions. In summary, this study lays the foundation for exploration of future control strategies for autonomous hip exoskeletons designed to assist load carriers. Further, we also plan to conduct future research to explore the effect of this type of assistance on different populations.

## Additional file

Additional file 1: Table S1. Questionnaire results indicated participants’ perceptions on assistive conditions on a visual analogue scale from 0 to 10. Q1: “How comfortable was this active condition?” Zero indicates unbearable and 10 indicates extremely comfortable. Q2: “How did you perceive the effect of the exosuit?” Zero indicates walking is impossible and 10 indicates walking is effortless. Early-start-early-peak (ESEP), early-start-late-peak (ESLP), late-start-early-peak (LSEP), late-start-late-peak (LSLP). **Table S2**. Spatiotemporal parameters. Early-start-early-peak (ESEP), early-start-late-peak (ESLP), late-start-early-peak (LSEP), late-start-late-peak (LSLP). Data are means ± SEM. (PDF 156 kb)

## Abbreviations

ANOVA: Analysis of variance
BF: Biceps femoris
EMG: Electromyographic
ESEP: Early-start-early-peak
ESLP: Early-start-late-peak
GM: Gluteus maximum
GRFs: Ground reaction forces
IMU: Inertial measurement unit
LSEP: Late-start-early-peak
LSLP: Late-start-late-peak
MG: Medial gastrocnemius
RF: Rectus femoris
RMS: Root mean square
SEM: Standard error of the mean
SOL: Soleus
VM: Vastus medialis
