# Supplementary material for: Effect of timing of hip extension assistance during loaded walking with a soft exosuit
Source: J Neuroeng Rehabil. 2016 Oct 3;13:87. doi: 10.1186/s12984-016-0196-8 (PMC5048481; doi:10.1186/s12984-016-0196-8)
Supplement: Additional file 1: Table S1. — Questionnaire results indicated participants’ perceptions on assistive conditions on a visual analogue scale from 0 to 10. Q1: “How comfortable was this active condition?” Zero indicates unbearable and 10 indicates extremely comfortable. Q2: “How did you perceive the effect of the exosuit?” Zero indicates walking is impossible and 10 indicates walking is effortless. Early-start-early-peak (ESEP), early-start-late-peak (ESLP), late-start-early-peak (LSEP), late-start-late-peak (LSLP). Table S2. Spatiotemporal parameters. Early-start-early-peak (ESEP), early-start-late-peak (ESLP), late-start-early-peak (LSEP), late-start-late-peak (LSLP). Data are means ± SEM. (PDF 156 kb) [file 12984_2016_196_MOESM1_ESM.pdf]

Additional files

Additional file 1: Table S1.

Table S1. Questionnaire results indicated participants' perceptions on assistive conditions on a visual analogue scale from 0 to 10. Q1: "How comfortable was this active condition?" Zero indicates unbearable and 10 indicates extremely comfortable. Q2: "How did you perceive the effect of the exosuit?" Zero indicates walking is impossible and 10 indicates walking is effortless. Early-start-early-peak (ESEP), early-start-late-peak (ESLP), late-start-early-peak (LSEP), late-start-late-peak (LSLP).

	Training Day				Testing Day			
	ESEP	ESLP	LSEP	LSLP	ESEP	ESLP	LSEP	LSLP
**Q1**	6.0 ± 0.57	6.5 ± 0.54	6.7 ± 0.24	5.6 ± 0.28	6.1 ± 0.31	5.5 ± 0.36	6.0 ± 0.29	6.2 ± 0.28
**Q2**	5.3 ± 0.68	5.9 ± 0.55	5.9 ± 0.57	5.1± 0.41	5.7 ± 0.46	5.1 ± 0.44	5.9 ± 0.41	5.6 ± 0.50

Additional file 1: Table S2.

Table S2. Spatiotemporal parameters. Early-start-early-peak (ESEP), early-start-late-peak (ESLP), late-start-early-peak (LSEP), late-start-late-peak (LSLP). Data are means ± SEM.

	Stride length (m)	Stride Frequency (Hz)	Stance Time (s)	Swing Time (s)	Duty Factor (%)
**Unpowered**	1.528 ± 0.024	0.983 ± 0.015	0.679 ± 0.011	0.340 ± 0.007	66.7 ± 0.3
**ESEP**	1.512 ± 0.021	0.993 ± 0.013	0.671 ± 0.008	0.337 ± 0.006	66.5 ± 0.2
**ESLP**	1.520 ± 0.023	0.989 ± 0.014	0.674 ± 0.009	0.339 ± 0.006	66.5 ± 0.2
**LSEP**	1.512 ± 0.020	0.993 ± 0.013	0.672 ± 0.008	0.336 ± 0.007	66.7 ± 0.3
**LSLP**	1.519 ± 0.023	0.989 ± 0.015	0.678 ± 0.010	0.335 ± 0.006	66.9 ± 0.3
